# Alumni Association of the Chicago Medical College

**Published:** 1872-03-01

**Authors:** 


### ALUMNI ASSOCIATION OF THE CHICAGO MEDICAL COLLEGE.

The fifth anniversary meeting of this Association was held at 10 a. m., March 12, in the College Hall.

A larger number than usual were present. Dr. Norman Bridge, President, in the chair.

No prize essays were presented this year. Dr. Brummund moved that two prizes be offered the ensuing year—one of $100, and a second of $50—to those members of the Association who shall present the first and second best essays on any medical or surgical topic, and that the committee on prize essays—Drs. N. S. Davis, Edmund Andrews, H. A. Johnson and Walter Hay—be requested to serve another year. It was moved by Dr. H. P. Merriman that the President's address be published in the Chicago MEDICAL EXAMINER, and that copies sufficient to supply the members be printed in pamphlet form.

Dr. Wm. Dougal moved that the Necrologist's report be included. Carried.

Drs. N. S. Davis and Lyman Ware each donate $50 toward the sums offered as prizes.

Officers for the ensuing year: Dr. Lyman Ware, President, class '66, Chicago, Ill.; Dr. Peter Brummund, Vice-President, class '68, Joliet, Ill.; Dr. M. P. Hatfield, 2d Vice-President, class '72, Chicago, Ill.; Dr. Norman Bridge, Necrologist, class '68, Chicago, Ill.; Dr. S. A. McWilliams, permanent Secretary and Treasurer, 125 Eighteenth street, Chicago, Illinois.

